# Induction of apoptosis and ganoderic acid biosynthesis by cAMP signaling in *Ganoderma lucidum*

**DOI:** 10.1038/s41598-017-00281-x

**Published:** 2017-03-23

**Authors:** Bang-Jau You, Ni Tien, Miin-Huey Lee, Bo-Ying Bao, Yih-Shyuan Wu, Tsung-Chi Hu, Hong-Zin Lee

**Affiliations:** 1 Department of Chinese Pharmaceutical Sciences and Chinese Medicine Resources, China Medical University, Taichung, Taiwan; 2 Department of Laboratory Medicine, China Medical University Hospital, Taichung, Taiwan; 3 Department of Plant Pathology, National Chung-Hsing University, Taichung, Taiwan; 4 Department of Pharmacy, China Medical University, Taichung, Taiwan

## Abstract

Apoptosis is an essential physiological process that controls many important biological functions. However, apoptosis signaling in relation to secondary metabolite biosynthesis in plants and fungi remains a mystery. The fungus *Ganoderma lucidum* is a popular herbal medicine worldwide, but the biosynthetic regulation of its active ingredients (ganoderic acids, GAs) is poorly understood. We investigated the role of 3′,5′-cyclic adenosine monophosphate (cAMP) signaling in fungal apoptosis and GA biosynthesis in *G. lucidum*. Two phosphodiesterase inhibitors (caffeine and 3-isobutyl-1-methylxanthine, IBMX) and an adenylate cyclase activator (sodium fluoride, NaF) were used to increase intracellular cAMP levels. Fungal apoptosis was identified by terminal deoxynucleotidyl transferase mediated dUTP nick end labeling (TUNEL) assay and a condensed nuclear morphology. Our results showed that GA production and fungal apoptosis were induced when the mycelium was treated with NaF, caffeine, or cAMP/IBMX. Downregulation of squalene synthase and lanosterol synthase gene expression by cAMP was detected in the presence of these chemicals, which indicates that these two genes are not critical for GA induction. Transcriptome analysis indicated that mitochondria might play an important role in cAMP-induced apoptosis and GA biosynthesis. To the best of our knowledge, this is the first report to reveal that cAMP signaling induces apoptosis and secondary metabolite production in fungi.

## Introduction

*Ganoderma lucidum*, a traditional Chinese herb, has been used to treat various human diseases such as cancer, inflammation, chronic hepatitis, heart disease, and hypertension for thousands years in Asia^1^. In addition, *G. lucidum* is also used in Asia as a tonic to enhance health, improve immunity, and delay senility. Ganoderic acids (GAs), a type of triterpenoids, are one of the major active ingredients of *G. lucidum*. The triterpenoids from *G. lucidum* exhibit a range of pharmacological activities including anticancer, antioxidant, hepatoprotective, hypocholesterolemic, and antiplatelet aggregation activities^1, 2^. Till date, more than 130 triterpenoids with different structural features have been identified from *G. lucidum* ^1, 3^. Recently, the genome sequence of *G. lucidum* has been published^4^ and a gene silencing approach has become available to knock down specific genes^5^. Thus, *G. lucidum* has the potential to be used as a model system for studying the regulation of secondary metabolite biosynthesis in Chinese herbal medicines.

The triterpenoid compounds have been proposed to be synthesized by the mevalonate/isoprenoid pathway, wherein mevalonate and isopentenyl pyrophosphate synthesized from acetyl CoA generate farnesyl diphosphate^6, 7^. In parallel, squalene and lanosterol have been proposed to be synthesized via pathways involving the enzymes squalene synthase (SQS) and lanosterol synthase (LS), respectively^8, 9^. A series of reduction, oxidation, acetylation, and hydroxylation reactions involving the products of the above pathways then form the various GAs^10^. However, the regulation of GA biosynthesis still remains largely unexplored.

The physiological roles of apoptosis have been well investigated in a range of organisms in different contexts, but its effects on secondary metabolite biosynthesis have not been explored much. Fungal elicitors have been shown to induce apoptosis and secondary metabolite biosynthesis in the plants *Artemisia annua*, *Taxus chinensis*, and *Cupressus lusitanica* ^11–13^. Our previous study has indicated that aspirin is able to induce apoptosis and GA production in *G. lucidum* ^14^. However, the mechanisms controlling this phenomenon remain unknown, as do the mechanisms that coordinate apoptosis and secondary metabolite biosynthesis. In addition, the regulation of GA biosynthesis and apoptosis in *G. lucidum* is poorly understood. In this study, the role of 3′,5′-cyclic adenosine monophosphate (cAMP) signaling in apoptosis and GA biosynthesis was investigated. Cells of the fungus *G. lucidum* were incubated with cAMP or various drugs that are able to activate adenylyl cyclase activity or inhibit phosphodiesterase activity. Apoptosis was evaluated by terminal deoxynucleotidyl transferase mediated dUTP nick end labeling (TUNEL) assay. GA production and the expression of various GA biosynthetic genes, including SQS and LS were measured. Transcriptome analysis was further conducted to investigate the global transcriptional changes associated with cAMP-induced apoptosis and GA biosynthesis.

## Results

### cAMP signaling induces apoptosis in the fungus *G. lucidum*

**Figure 1:**
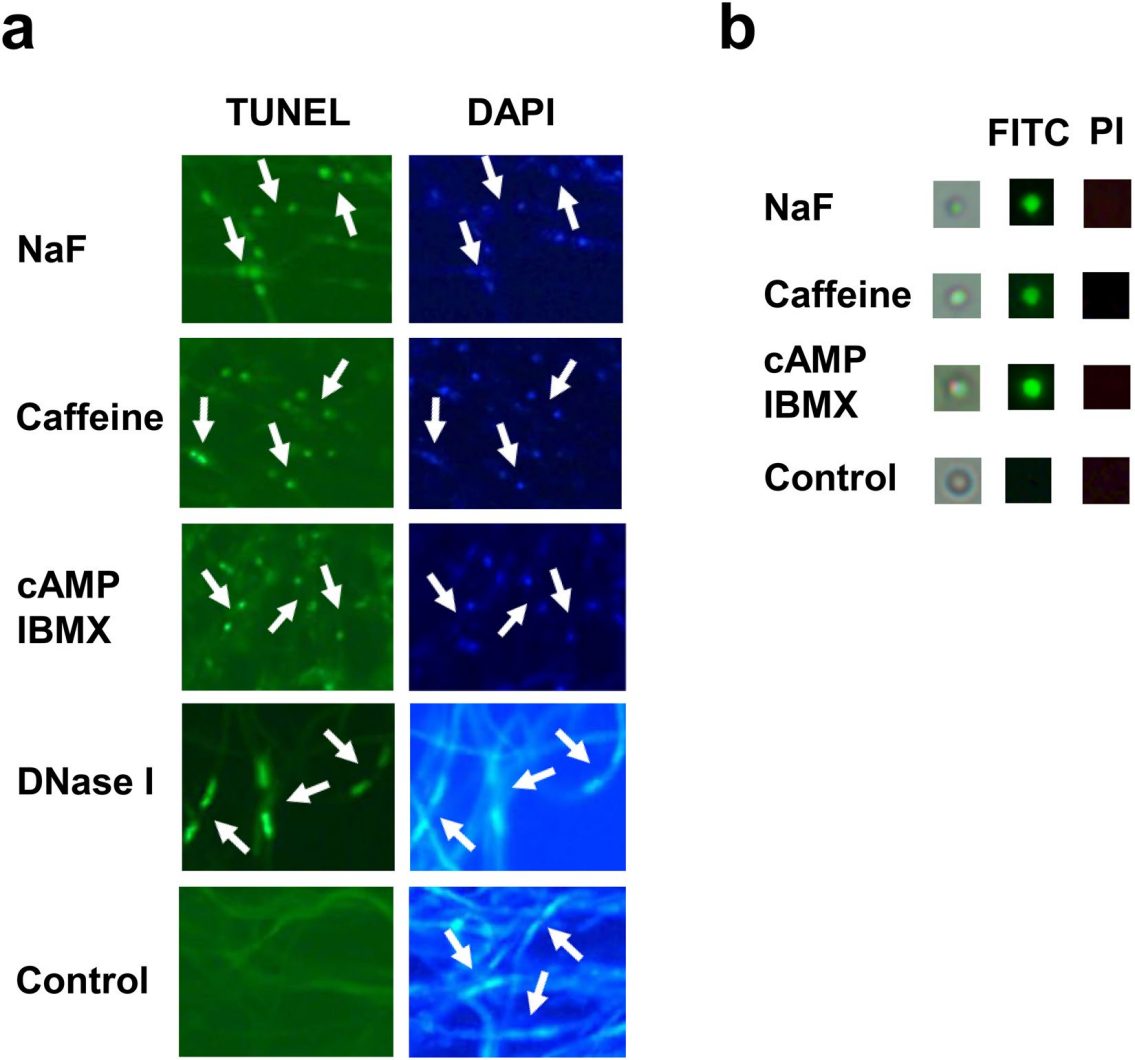
Apoptosis induction in *Ganoderma lucidum*. Fungal mycelium was incubated with 20 mM NaF, 80 mM caffeine, or 40 mM cAMP/15 mM IBMX for 16 h prior to staining. (**a**) DNA fragmentation was assessed by TUNEL assay. DAPI staining was used to locate the nucleus and assess the nuclear morphology of the fungal cells. The arrows indicate the two nuclei present in each fungal cell. Untreated fungal cells did not show any fluorescence signal in the TUNEL assay. To further confirm the relevance of the nuclear morphology of the fungal cells, mycelium was pretreated with DNase I to induce DNA breaks and then subjected to TUNEL assay and DAPI staining. (**b**) Isolated protoplasts were stained with FITC-conjugated annexin V and propidium iodide (PI).

To investigate the role of cAMP signaling in fungal apoptosis, the mycelium of *G. lucidum* was treated with caffeine and NaF. Caffeine is an inhibitor of phosphodiesterase, while NaF is an activator of adenylate cyclase, and both of these drugs have been used to increase cytosolic cAMP levels in fungi^15–17^. Fungal apoptosis was identified by the presence of double-stranded DNA degradation and changes in nuclear morphology detected by the TUNEL assay and DAPI (4′,6-diamidino-2-phenylindole) staining, respectively. Typical results are shown in Fig. 1a. No fluorescent signal was observed by TUNEL staining in untreated (control) fungal cells, indicating that the genomic DNA in these cells was intact. Further, nuclear staining with DAPI revealed that the control cells had a normal nuclear morphology. A few fungal cells were detected as being positive for TUNEL staining when the mycelium was treated with 20 mM caffeine. At higher levels of caffeine (40 and 80 mM), a large number of TUNEL-positive cells with a condensed nuclear morphology were detected. Apoptotic fungal cells with condensed nuclei were also observed when the fungal cells were incubated in 10 and 20 mM NaF. To further evaluate the effect of cAMP signaling on apoptosis in *G. lucidum*, the fungal mycelium was treated with cAMP and IBMX, a phosphodiesterase inhibitor. As shown in Fig. 1a, co-treatment with cAMP and IBMX produced results to similar those obtained with caffeine and NaF, showing the induction of apoptosis.

Phosphatidylserine (PS) is located in the inner leaflet of the plasma membrane and its externalization is a marker for apoptosis. Annexin V has a high affinity for PS and can be used to detect PS externalization. After treating the fungal mycelium with NaF, caffeine, or cAMP/ IBMXP, the protoplasts were isolated and stained with FITC (fluorescein isothiocyanate)-conjugated annexin V and propidium iodide (PI). Typical results are shown in Fig. 1b. No fluorescent signal was observed in the control protoplasts thus stained. The fungal protoplasts derived from mycelium treated with NaF, caffeine, or cAMP/IBMXP showed green fluorescence, indicating positive FITC-annexin V staining. However, no fluorescent signal was seen when the protoplasts were stained with PI. This data suggests that treatment with NaF, caffeine, or cAMP/IBMX induced apoptosis in the fungus *G. lucidum* (Fig. 1b).

### Effects of caffeine on fungal biomass and production of ganoderic acids

**Figure 2:**
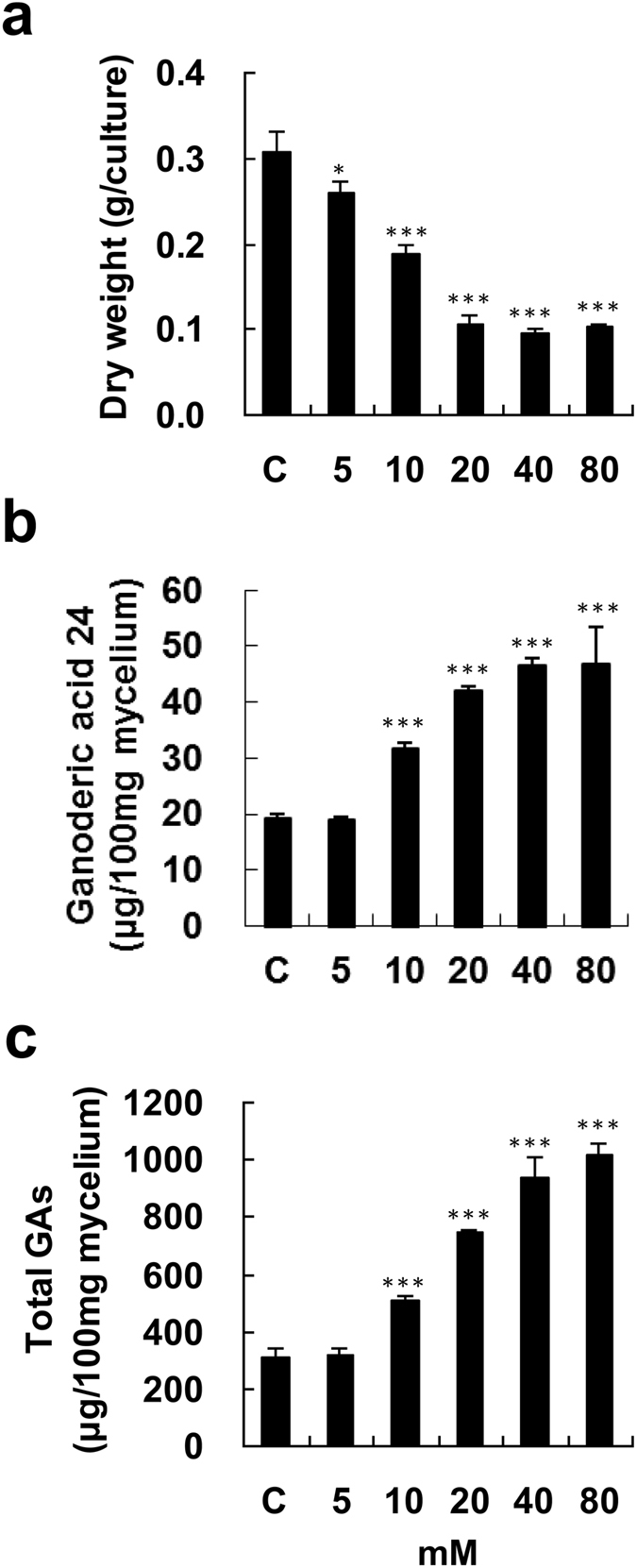
Effect of caffeine on fungal biomass and production of ganoderic acids in *Ganoderma lucidum*. Four-day-old fungal mycelium was cultured on potato dextrose agar (PDA) in 9-cm diameter petri dishes, and then incubated with 5–80 mM caffeine in 25 ml potato dextrose broth (PDB) for 4 days. Dried mycelium was used to measure fungal biomass (**a**). Both lanosta-7,9(11), 24-trien-3α-o1-26-oic acid (ganoderic acid 24) (**b**), and total ganoderic acids (GAs) (**c**) were measured by HPLC. The means of three independent samples with standard deviations are presented. *p < 0.05, **p < 0.01, ***p < 0.001 as compared with the control group.

**Figure 3:**
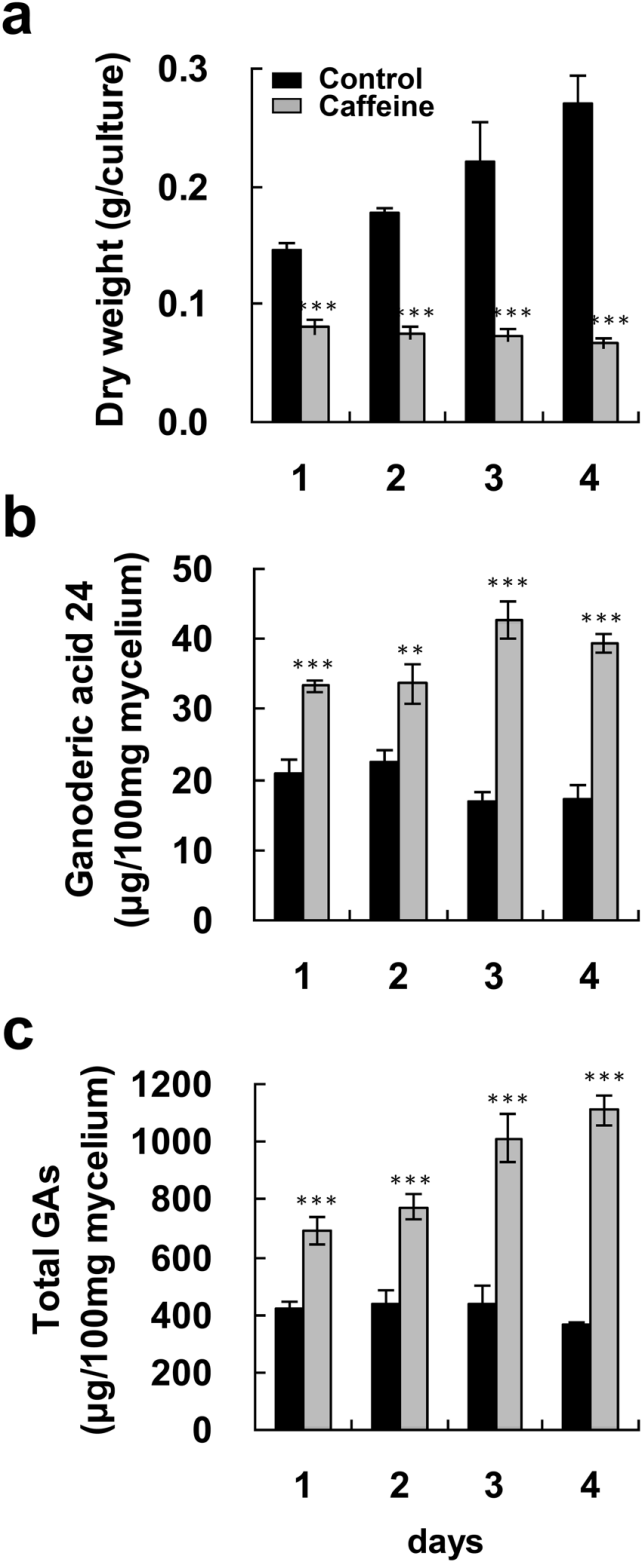
Time-course of fungal biomass and ganoderic acid production by *Ganoderma lucidum* after treatment with caffeine. Four-day-old fungal mycelium was cultured on potato dextrose agar (PDA) in 9-cm diameter petri dishes and then incubated with 80 mM caffeine in 25 ml potato dextrose broth (PDB) for 1–4 days. Dried mycelium was used to measure fungal biomass (**a**). Both lanosta-7,9(11), 24-trien-3α-o1-26-oic acid (ganoderic acid 24) (**b**), and total ganoderic acids (GAs) (**c**) were measured by HPLC. The means of three independent samples with standard deviations are presented. *p < 0.05, **p < 0.01, ***p < 0.001 as compared with the control group.

Fungal mycelium was incubated with 5–80 mM caffeine for 4 days in order to evaluate its effects on biomass and GA production. A slight reduction in biomass was observed when the mycelium was incubated with 5 mM caffeine, whereas treatment with 20–80 mM caffeine greatly reduced biomass (Fig. 2a). Lanosta-7,9(11), 24-trien-3α-o1-26-oic acid (ganoderic acid 24 (GA24)) and total GA production were also evaluated by high-performance liquid chromatography (HPLC). With an increase in the concentration of caffeine surrounding the mycelium pieces from 10 to 80 mM, there was a gradual increase in the levels of GA24 and total GAs (Fig. 2b). The maximum levels of GA24 and total GAs, 2.43-fold and 3.26-fold higher than those in the control, respectively, were observed at the highest drug concentration. A time-course study of GA production upon treatment with 80 mM caffeine was also done. After one-day incubation with caffeine, GA24 and total GAs were significantly increased. The maximum levels of GA24 and total GAs, reached after 3 and 4 days incubation, were 42.64 and 1109.14 μg/100 mg mycelium, respectively (Fig. 3).

### Effects of NaF on fungal biomass and production of ganoderic acids

**Figure 4:**
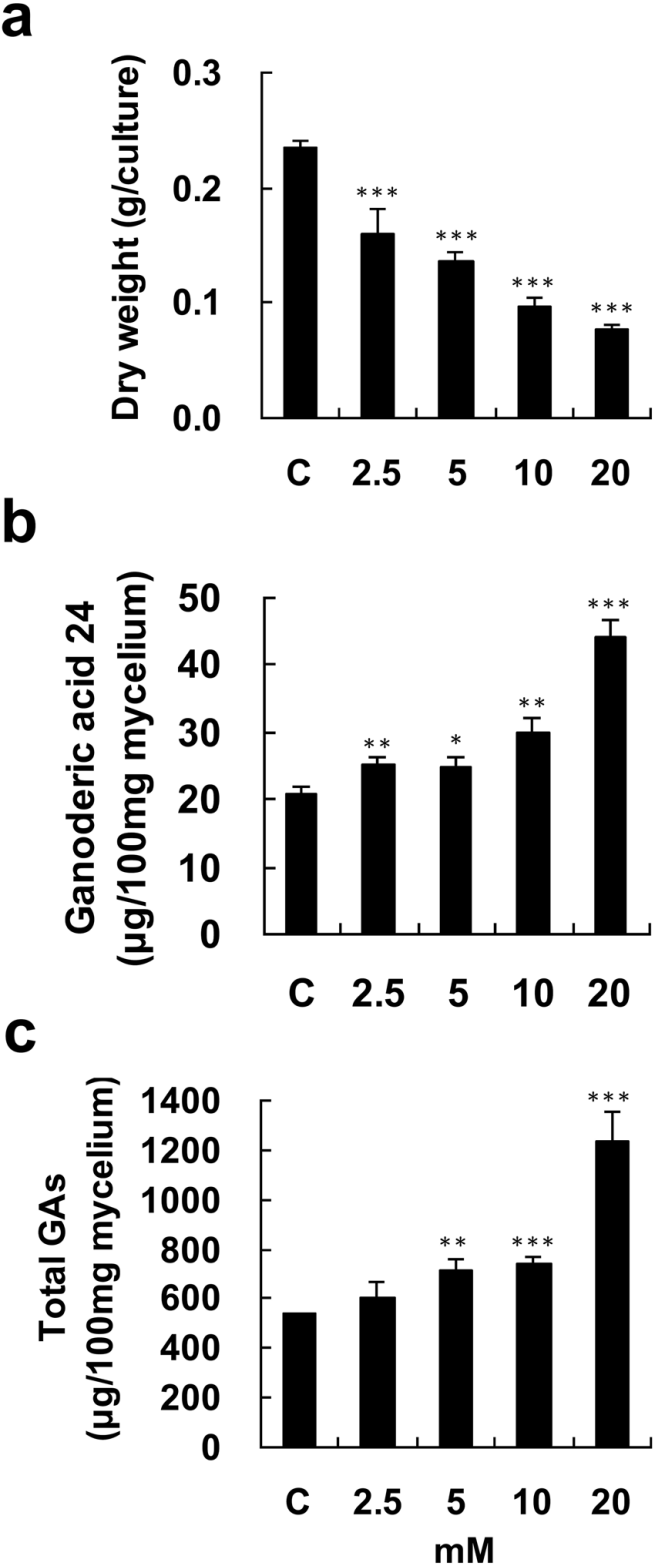
Effect of NaF on fungal biomass and production of ganoderic acids by *Ganoderma lucidum*. Four-day-old fungal mycelium was cultured on potato dextrose agar (PDA) in 9-cm diameter petri dishes and then incubated with 2.5–20 mM NaF in 25 ml potato dextrose broth (PDB) for 4 days. Dried mycelium was used to measure fungal biomass (**a**). Both lanosta-7,9(11), 24-trien-3α-o1-26-oic acid (ganoderic acid 24) (**b**), and total ganoderic acids (GAs) (**c**) were measured by HPLC. The means of three independent samples with standard deviations are presented. *p < 0.05, **p < 0.01, ***p < 0.001 as compared with the control group.

**Figure 5:**
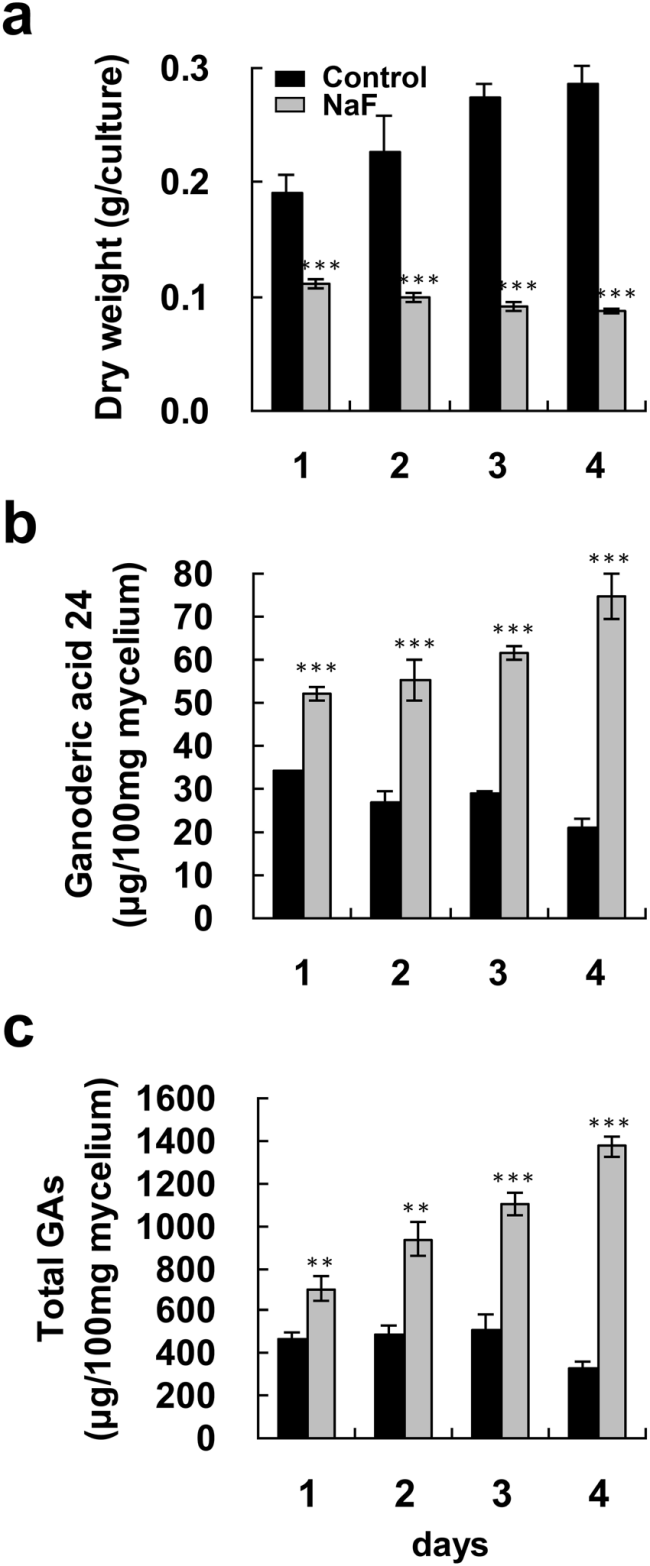
Time-course of fungal biomass and ganoderic acid production by *Ganoderma lucidum* treated with NaF. Four-day-old fungal mycelium was cultured on potato dextrose agar (PDA) in 9-cm diameter petri dishes and then incubated with 20 mM NaF in 25 ml potato dextrose broth (PDB) for 1–4 days. Dried mycelium was used to measure fungal biomass (**a**). Both lanosta-7,9(11), 24-trien-3α-o1-26-oic acid (ganoderic acid 24) (**b**), and total ganoderic acids (GAs) (**c**) were measured by HPLC. The means of three independent samples with standard deviations are presented. *p < 0.05, **p < 0.01, ***p < 0.001 as compared with the control group.

When 2.5–20 mM NaF was used to treat fungal mycelium for 4 days, it was found that the biomass of *G. lucidum* gradually decreased as the NaF concentration increased (Fig. 4). Administration of 2.5–10 mM NaF only slightly enhanced GA24 and total GA production, whereas incubation with 20 mM NaF significantly increased the levels of GA24 and total GAs to 2.1-fold and 2.31-fold higher than their levels in the control, respectively (Fig. 4b,c). A time-course study of GA24 and total GAs upon incubation with 20 mM NaF was carried out. There was significant enhancement of GA24 and total GA production after one-day incubation with NaF, while a longer incubation yielded even higher amounts of GA24 and total GAs. The highest levels of GA24 and total GAs were 74.81 and 1372.42 μg/100 mg mycelium, respectively, after 4 days of incubation (Fig. 5).

### Effects of cAMP and IBMX on fungal biomass and production of ganoderic acids

**Figure 6:**
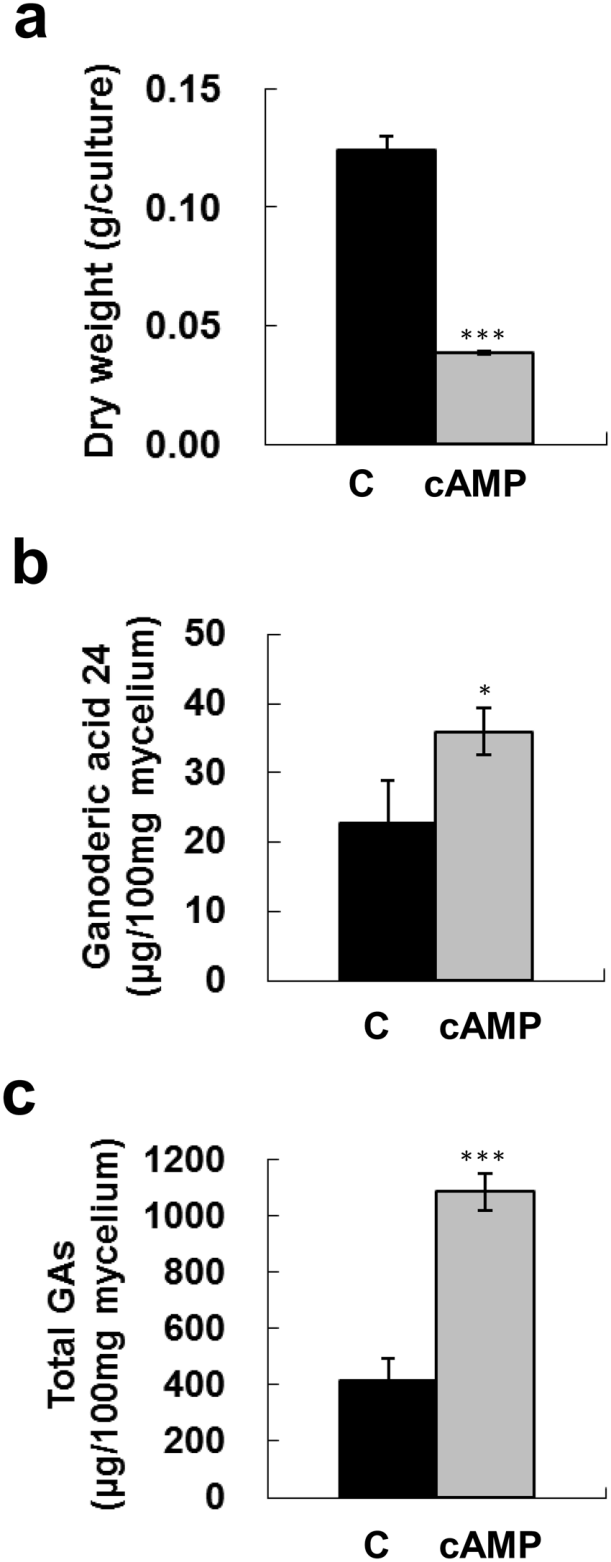
Effect of cAMP and IBMX co-treatment on fungal biomass and production of ganoderic acids. Four-day-old fungal mycelium was cultured on potato dextrose agar (PDA) in 5.5-cm diameter petri dishes and then incubated with 40 mM cAMP and 15 mM IBMX in potato dextrose broth (PDB) for 4 days. Dried mycelium was used to measure fungal biomass (**a**). Lanosta-7,9(11), 24-trien-3α-o1-26-oic acid (ganoderic acid 24) (**b**), and total ganoderic acids (GAs) (**c**) were measured by HPLC. The means of three independent samples with standard deviations are presented. *p < 0.05, **p < 0.01, ***p < 0.001 as compared with the control group.

Fungal mycelium was treated simultaneously with cAMP and IBMX, a phosphodiesterase inhibitor, to further investigate the effect of cAMP signaling on GA production. Fungal mycelia from a 4-day-old culture were incubated with 40 mM cAMP and 15 mM IBMX for 4 days. Treatment with cAMP/IBMX significantly decreased biomass, but enhanced GA24 and total GA production by 1.58-fold and 2.61-fold, respectively, as compared to their levels in the control (Fig. 6). These findings further support the hypothesis that cAMP signaling controls GA biosynthesis.

### Gene expression of squalene synthase and lanosterol synthase

**Figure 7:**
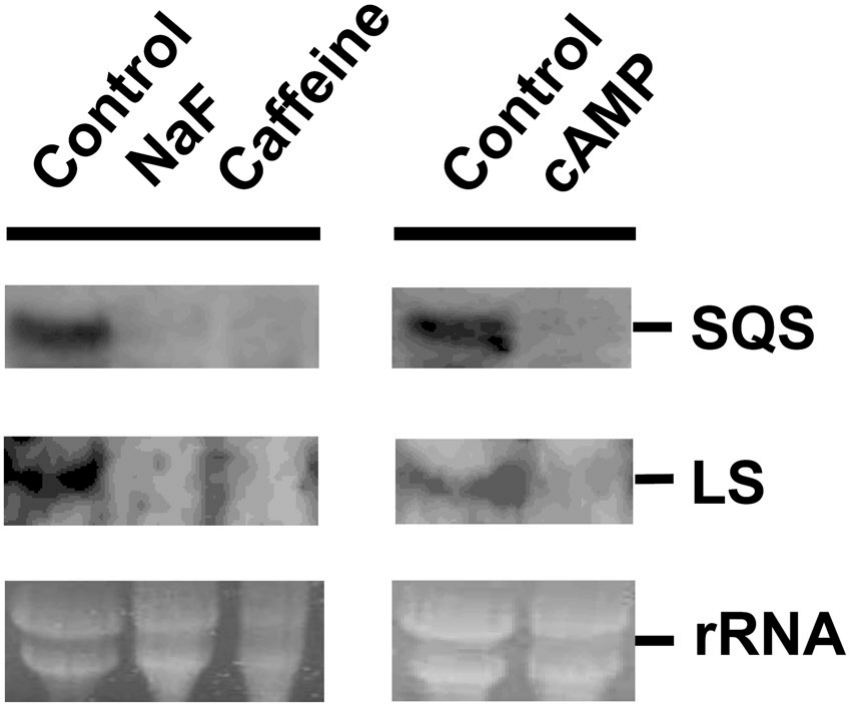
Effect of cAMP on the mRNA expression levels of squalene synthase (SQS) and lanosterol synthase (LS) in *Ganoderma lucidum*. *G. lucidum* mycelium was incubated with 40 mM caffeine, 10 mM NaF, or 40 mM cAMP/15 mM IBMX for 12 h. Northern blotting was then performed to detect the expression of the SQS and LS genes. Gels stained with ethidium bromide are shown to indicate the relative total RNA loading.

We evaluated the effect of cAMP signaling on the expression of two GA biosynthetic genes, SQS and LS, using northern blotting analysis. The findings showed that treatment with cAMP reduced the transcription of the SQS and LS genes (Fig. 7). These findings are in agreement with previous studies which detected a lower expression of SQS and LS upon treatment with a high dose of reactive oxygen species (ROS) or aspirin^14, 18^.

### Transcriptome sequencing for cAMP-induced differential gene expression

To investigate the global transcriptional changes associated with cAMP-induced apoptosis and GA biosynthesis, transcriptome sequencing was performed. We found that 5836 genes were expressed differently between the control and cAMP-treated mycelium. Out of these, 3825 genes were upregulated and 2011 genes were downregulated in the cAMP-treated mycelium (Supplementary Table 1).

### Gene ontology and KEGG pathway analysis of differentially expressed genes

**Figure 8:**
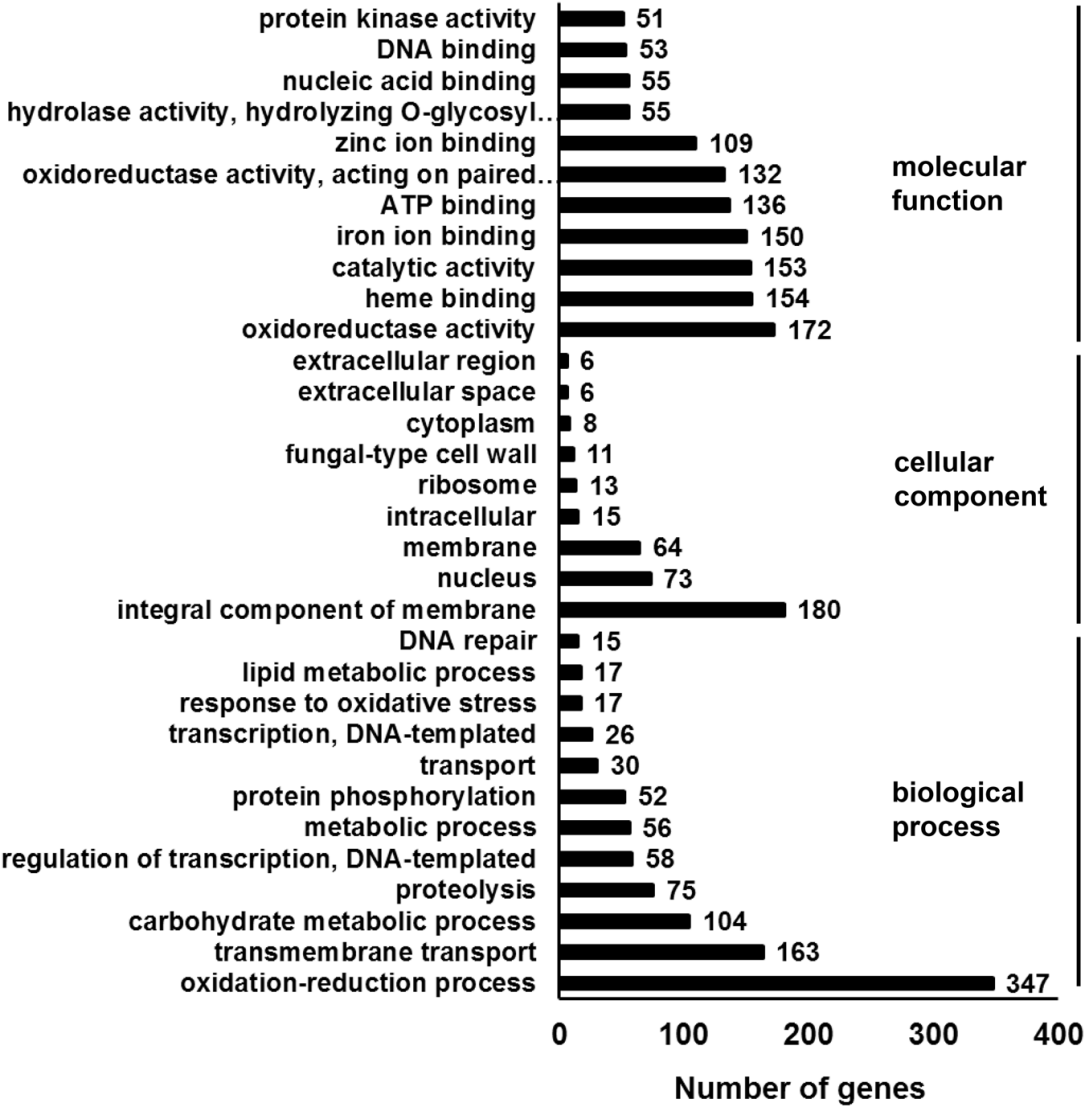
Gene Ontology analysis. The fungal mycelium was incubated with cAMP and IBMX. Transcriptome sequencing was performed to identify differentially expressed genes between the control and cAMP-treated mycelium. The differentially expressed genes were further mapped to the gene ontology (GO) database. Three main categories (biological process, cellular component, and molecular function) are presented.

Differentially expressed genes were characterized into 449 Gene Ontology (GO) terms and most of them fell into the “molecular function” domain. The highest-enriched GO terms in the “biological process” domain were oxidation-reduction process, transmembrane transport, and carbohydrate metabolic process (Fig. 8). In the “molecular function” domain, the GO terms with the highest enrichment were oxidoreductase activity, heme binding, and catalytic activity. Our results also showed that the expression of the cAMP phosphodiesterase gene was significantly enhanced in cAMP-treated fungi, indicating that their cAMP decomposition was affected.

**Figure 9:**
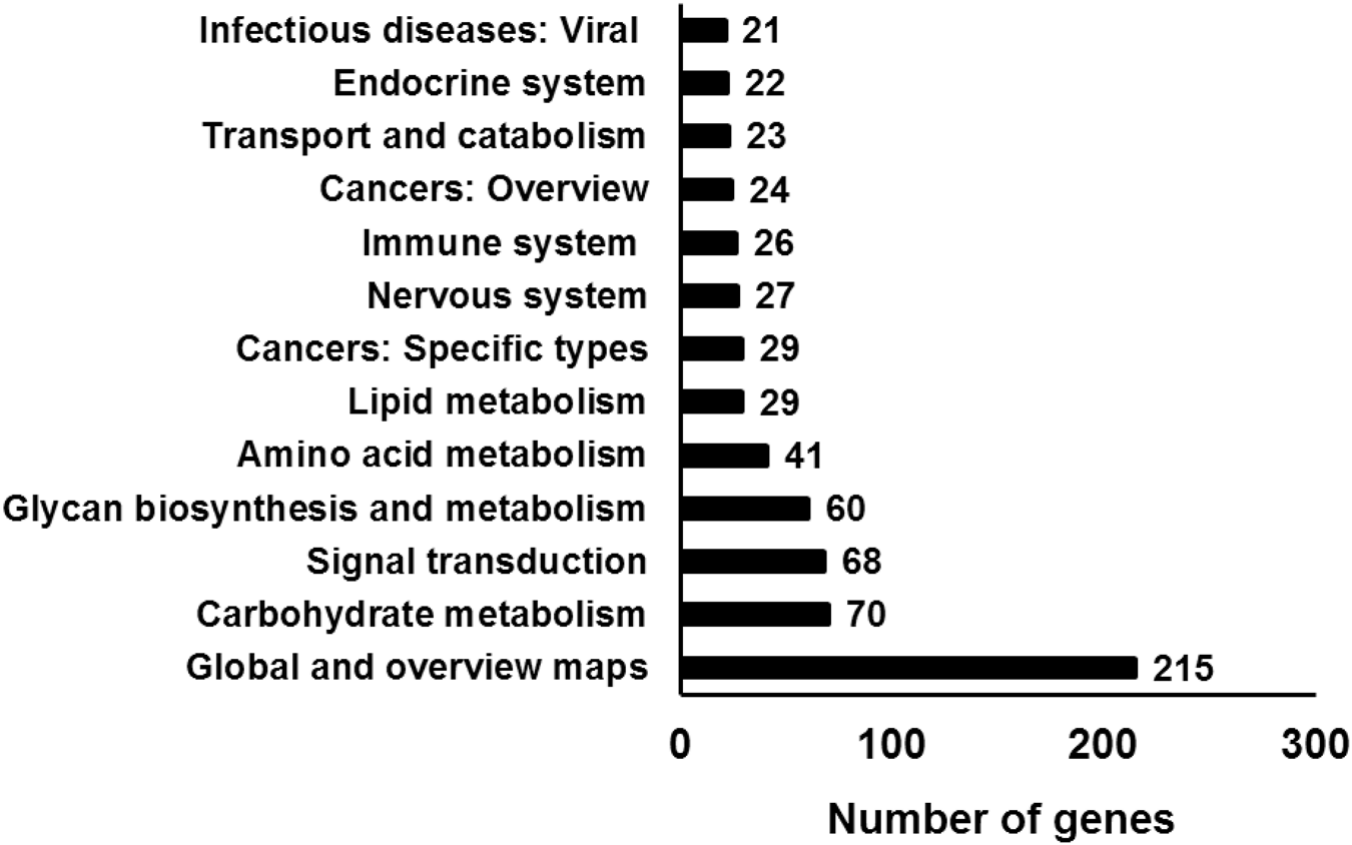
KEGG pathway analysis. The fungal mycelium was incubated with cAMP and IBMX. Transcriptome sequencing was performed to identify differentially expressed genes between the control and cAMP-treated mycelium. The differentially expressed genes were further mapped to the KEGG database. The major categories of differentially expressed genes are presented.

The differentially expressed genes were further mapped to the Kyoto Encyclopedia of Genes and Genomes (KEGG) pathway (Fig. 9). These genes were grouped majorly into the categories: “global and overview maps” and “carbohydrate metabolism”. cAMP incubation also affected gene expression in the “signal transduction” category, which included the MAPK, sphingolipid, FoxO, TNF, PI3K-AKT, and mTOR signaling pathways.

## Discussion

Apoptosis plays a range of important functions in animals, plants, and microorganisms. For example, apoptosis controls embryonic development, tissue homeostasis, immune system function, and atrophy in mammals^19^. Any dysfunction affecting apoptosis can cause a number of diseases including cancer, autoimmunity, and neurodegenerative disorders^20^. In plants, apoptosis-like programmed cell death is quite similar to apoptosis in animals, and includes similar features of DNA degradation, chromatin condensation, and caspase activation. Apoptosis in plants is involved in defense reactions, plant development, and stress response^21^. Among fungal microorganisms, the long-term survival of yeast colonies and the replicative lifespan in yeast are regulated by apoptosis^22, 23^. Until now, very few studies have focused on the mechanisms that coordinate apoptosis and secondary metabolite biosynthesis in plants and fungi. The biosynthesis of taxol, β-thujaplicin, and artemisinin in plants have been shown to be induced during apoptosis caused by biotic elicitors from the fungal cell wall^11–13^. Our previous study indicated that aspirin is able to induce both apoptosis and GA production in *G. lucidum* ^14^. However, the cellular signaling pathways that control both apoptosis and secondary metabolite biosynthesis in plants and fungi remain enigmatic. Here, using cAMP, an adenynyl cyclase activator, and phosphodiesterase inhibitors we showed that cAMP signaling regulated apoptosis and GA biosynthesis in *G. lucidum*. To the best of our knowledge, this study is the first to uncover a mechanism for controlling apoptosis and secondary metabolite biosynthesis in a fungus. In a plant study, Zhao *et al*. have indicated that nitric oxide (NO) mediates β-thujaplicin production and apoptosis in *Cupressus lusitanica* cells treated with a yeast elicitor^13^. Whether cAMP signaling also mediates apoptosis and secondary metabolite biosynthesis in plants remains unknown.

*G. lucidum* is an important Chinese medicine widely used worldwide. However, the regulation of GA biosynthesis in *G. lucidum* is poorly understood. Previous work by Zhong *et al*. and our lab have demonstrated that ROS and Ca^2+^ are able to regulate GA biosynthesis^18, 24, 25^. Sodium and manganese have been shown to trigger Ca^2+^ signaling, which, in turn, leads to an enhancement of GA production^26, 27^. Recently, Mu *et al*. have demonstrated that nicotinamide adenine dinucleotide phosphate (NADPH) oxidase is able to regulate GA biosynthesis by producing ROS, which, in turn, affect the Ca^2+^ signaling pathway^28^. The present study is the first to indicate that cAMP is also able to regulate GA biosynthesis in *G. lucidum*. Several other studies have also shown that cAMP signaling can have diverse effects on secondary metabolite biosynthesis in fungi. Examples of such secondary metabolites include fusarubin^29^, bikaverin^29, 30^, sterigmatocystin^31^, lovastatin^32^, citrinin^32^, gibberellins^33^, and carotenoids^33^.

Fungal apoptosis is an emerging field compared to the well-established field of animal apoptosis. The signaling pathways controlling apoptosis in *G. lucidum* are yet unknown. Our study has revealed that cAMP is able to induce apoptosis in *G. lucidum* in a similar manner to the cAMP-mediated induction of apoptosis in mammals^34, 35^. Such a similarity in the mechanisms controlling apoptosis in fungi and mammals is very interesting in an evolutionary context. A number of previous studies have suggested that cAMP signaling is involved in fungal apoptosis. For example, in *Candida albicans*, mutations in the Ras/cAMP/protein kinase A (PKA) signaling pathway genes suppressed apoptosis caused by acetic acid and hydrogen peroxide^16^. Similarly, exogenous dibutyryl-cAMP, a cAMP analog, inhibited lovastatin-induced apoptosis in the fungus *Mucor racemosus* ^36^, and actin-mediated apoptosis was shown to be regulated by cAMP signaling in *Saccharomyces cerevisiae* ^37, 38^.

In mammals, the main downstream effectors of cAMP are PKA, exchange protein directly activated by cAMP (EPAC), and cyclic nucleotide-gated ion channels^39^. The cAMP/PKA pathway controls mitochondrial morphology, biogenesis, and energy metabolism via protein phosphorylation^40^. Moreover, activation of cAMP/PKA signaling resulted in overexpression of the oxidative phosphorylation complex proteins^41^. PKA is also the main effector that controls apoptosis in cAMP signaling. PKA phosphorylated pro-apoptotic proteins to induce the intrinsic apoptosis pathway^39^. Several studies indicated that PKA was also involved in ROS-mediated apoptosis^39^. The inappropriate activation of one of the PKA catalytic subunits induced mitochondrial dysfunction, ROS production, and apoptosis in yeast^42^. Our GO ontology analysis (Fig. 8) indicated that the genes differentially expressed in response to cAMP treatment belonged mainly to the categories: “oxidation-reduction process” and “oxidoreductase activity.” In addition, our transcriptome analysis indicated an increase in the expression of genes homologous to the oxidative phosphorylation complexes such as cytochrome c, cytochrome c oxidase subunits, mitochondrial-processing peptidase subunit beta, cytochrome b-c1 complex subunit 8, and ATP synthase subunits (Supplementary Table 2). These results suggest that mitochondria might play an important role in cAMP-induced apoptosis and GA biosynthesis observed in this study, and their role may be investigated in future studies. The sphingolipid^43, 44^, MAPK^45, 46^, and PI3K-Akt^47^ signaling pathways are also known to regulate apoptosis in mammals. In this study too, the exposure of cells to cAMP affected the expression of genes in the sphingolipid, MAPK, and PI3K-AKT signaling pathways (Supplementary Figures 1–3).

Transcription levels of key genes related to GA biosynthesis, including SQS, LS, and 3-hydroxy-3-methylglutaryl CoA reductase (HMGR) have been characterized in several studies. The expression of SQS, LS, and HMGR was enhanced in static liquid culture as compared with shaking culture^48^. Sodium, methyl jasmonate, phenobarbital, and acetic acid induced GA production and increased the expression of GA biosynthetic genes^26, 49–51^. However, our data showed that NaF, caffeine, and cAMP induced GA production but reduced the gene expression of SQS and LS. This is in agreement with our previous study, which showed that the induction of GA production by high doses of ROS and aspirin reduced the SQS and LS mRNA expression^14, 18^. Therefore, these findings support the idea that high doses of ROS, aspirin, and cAMP may upregulate the GA biosynthetic genes downstream of lanosterol biosynthesis, and this needs to be investigated further.

The production of secondary metabolites, such as GAs, is an important concern as it affects the commercial usefulness of medicinal fungi and plants. It takes a long time (~3–5 months) to produce the fruiting bodies of *G. lucidum*, therefore, studies investigating submerged cultures for improving GA production have gained a lot of attention recently^48, 52^. In addition, various inducers such as methyl jasmonate^49, 53^, phenobarbital^50^, manganese^27^, Ca^2+^ ^24^, and acetic acid^51^ have been found to enhance GA production. Our previous report^14^ and the current study are the first to identify a new approach to enhance GA production, whereby abiotic chemicals are used to induce apoptosis in fungi, using *G. lucidum* as a model. This approach to increase secondary metabolite production could be useful to the functional food and pharmaceutical industries for the large-scale production of these metabolites. However, further investigation is needed to determine whether abiotic chemical inducers such as cAMP can induce apoptosis in plants and other fungi, and whether this approach can be used to enhance their production of secondary metabolites.

## Conclusion

Apoptosis controls many important biological functions and has been studied extensively in animals. However, the regulatory signaling that controls apoptosis and secondary metabolite biosynthesis in plants and fungi has only been investigated to a very limited extent. Our study is the first to show that cAMP signaling controls apoptosis and secondary metabolite biosynthesis in the fungus, *G. lucidum*. In addition, we believe that the regulation of secondary metabolite biosynthesis is critical to the production of active compounds by many medicinal plants and fungi. The present findings indicate that there is an enhancement of secondary metabolite production upon induction of apoptosis by abiotic chemical inducers. We propose that this approach may be usefully extended to other medicinal fungi and plants. To summarize, this study provides insights into the regulation of triterpenoid biosynthesis in an important Chinese medicine, *G. lucidum*, and the interaction of the biosynthetic pathways with fungal apoptosis via cAMP signaling.

## Materials and Methods

### Fungal strain and culture conditions

*G. lucidum* strain BCRC 36111 was obtained from the Bioresource Collection and Research Center (Hsin Chu, Taiwan). The fungal mycelium was maintained on potato dextrose agar (PDA; Difco, Sparks, MD, USA) at 28 °C. For the induction of GA production, the fungus was cultured and treated with drugs as described in our previous study^14^. Fungal mycelium from a 7- to 10-day-old culture was smashed into fragments using a sterile blender and spread onto PDA overlaid with cellophane for 4 days. The fungal mycelium was then treated with caffeine, NaF, or cAMP/IBMX in flasks containing potato dextrose broth (PDB), and incubated for 1–4 days under continuous shaking at 100 rpm. Finally, the fungal mycelium was collected, dried, and subjected to analysis to measure GA production. All treatments were performed using at least three replicates and the experiments were repeated at least 3 times.

### TUNEL assay and nuclear staining

Fungal apoptosis was analyzed by TUNEL assay using an *In Situ* Cell Death Detection kit (Roche Applied Science, Indianapolis, IN, USA) as described in a previous study^14^. Fungal mycelium was treated with caffeine, NaF, or cAMP/IBMX for 16 h. The fungal cells were then fixed by treatment with 4% paraformaldehyde for 1 h, and the cell wall was digested with cell wall-degrading enzymes for 30 min. Cell permeabilization and staining procedures were performed according to the manufacturer’s guidelines. After TUNEL staining, DAPI (2 μg mL^−1^) was used to stain the fungal mycelium in order to detect the nuclei. The stained cells were imaged by fluorescence microscopy (IX70, Olympus, Tokyo, Japan). At least three independent experiments were performed.

### Annexin V Staining

To examine PS externalization, protoplasts of *G. lucidum* were stained with FITC-conjugated annexin V using Alexa Fluor® 488 Annexin V/Dead Cell Apoptosis Kit (Invitrogen, Eugene, OR, USA). Fungal mycelium was treated with NaF, caffeine, or cAMP/IBMX. The mycelium was then washed three times with sucrose buffer (0.6 M sucrose, 20 mM phosphate buffer, pH 5.8) and digested with 2% lysing enzyme in sucrose buffer at 30 °C for 2 h for protoplast release. The harvested protoplasts were washed and suspended in annexin V binding buffer (10 mM HEPES, 140 mM NaCl, 2.5 mM CaCl_2_, pH 7.4) containing 1 M sorbitol. Annexin V-FITC and PI were used to stain the protoplasts according to the manufacturer’s instructions.

### Quantitative analysis of ganoderic acids by HPLC

The quantification of GAs in samples of the fungal mycelium was conducted as described in previous studies^18, 54^. GAs were extracted from the fungal mycelium using methanol and then analyzed by HPLC. A pure standard of GA 24 was used to construct a calibration curve for calculating the production of GA24 and total GAs. Total GAs were calculated as the sum of the peak areas of compounds eluted from 5 to 50 min by HPLC analysis.

### Northern blotting analysis of squalene synthase and lanosterol synthase

For evaluating the gene expression of SQS and LS at the mRNA level, the corresponding DNA fragments were cloned from *G. lucidum* BCRC 36111^18, 54^, and northern blotting was performed using standard procedures. Trizol reagent (Invitrogen, Carlsbad, CA, USA) was used to extract fungal total RNA per the manufacturer’s protocol. The DNA probe for the SQS gene was amplified by PCR using the primers glssF263 (5′-TGGACACGATCGAAGATGACATGAC-3′) and glssR1492 (5′-GCCATCGTTTGTGGGATCGCACAGAA-3′), with the incorporation of digoxigenin-11-dUTP (Roche Applied Science). The primers gllsF1292 (5′-CGGCGTATCGGCACCAGACGAA-3′) and gllsR2105 (5′-TTCGGGTACGATATCGCGACGTTC-3′) were used to amplify the DNA probe for the LS coding region. Immunological detection of each northern blot was carried out using CDP-Star® chemiluminescent substrate according to the manufacturer’s instructions (Roche Applied Science). All experiments were conducted at least three times.

### Statistical analysis

Statistical analysis was performed using Microsoft Excel (Microsoft, Seattle, WA, USA). Student’s t-tests were conducted to evaluate any significant differences in GA production and biomass production between the control group and the various treatment groups. Statistical significance was expressed as: *p < 0.05, **p < 0.01, ***p < 0.001.

### RNA extraction and transcriptome sequencing

Fungal mycelium was treated with 40 mM cAMP and 15 mM IBMX for 12 h. Total RNA was extracted with TRIzol reagent using the standard protocol (Invitrogen, CA, USA) and DNase was used to remove potential DNA contamination. RNA purity and integrity were analyzed using the RNA Nano 6000 Assay Kit with the Bioanalyzer 2100 system (Agilent Technologies, CA, USA). Purified mRNA was fragmented and TruSeq Stranded mRNA Library Prep Kit (Illumia, San Diego, USA) was used for sequencing library construction per manufacturer’s protocols. Clustering of the index-coded samples was conducted on a cBot Cluster Generation System using TruSeq PE Cluster Kit v3-cBot-HS (Illumina) per manufacturer’s guidelines. The library products were then sequenced on a NextSeq 500 platform. For each sample, more than 23 M total reads were obtained. After sequencing, the adaptor sequence, low quality reads, and reads containing poly-N were removed to obtain clean reads using in-house Perl scripts. Q20, Q30, sequencing duplication, and GC content of the clean data were calculated. The clean datasets were used for subsequent analysis.

### De novo transcriptome assembly

De novo transcriptome assembly was carried out using a short read assembling program, Trinity (http://trinityrnaseq.sourceforge.net/). Trinity processes large volumes of RNA-seq reads using three independent software modules, *Inchworm*, *Chrysalis*, and *Butterfly* applied sequentially. The sequence data is partitioned into individual de Bruijn graphs that represent the transcriptional complexity at a given gene or locus. Each graph is processed independently to extract full-length splicing isoforms and to identify transcripts derived from paralogous genes^55^.

### Gene expression analysis and annotation

Abundance estimation methods were used to obtain transcript-level estimates of the count of RNA-Seq fragments derived from each transcript. The transcript levels were normalized using the “fragments per kilobase transcript length per million fragments mapped” (FPKM) metric. Normalization takes into account the transcript length, the number of reads mapped to the transcript, and the total number of reads mapped to any transcript. Differentially expressed transcripts were identified using the edgeR Bioconductor package^56^. For gene annotation, BLASTX alignment (E-value < 0.00001) was performed between the unigenes and the following databases: GO, KEGG^57, 58^, non-redundant (NR), and Swiss-Prot.

## Electronic supplementary material

Supplementary information

Supplementary Table 1

Supplementary Table 2

## References

[1] Paterson RR (2006). *Ganoderma*–a therapeutic fungal biofactory. Phytochemistry.

[2] Baby S, Johnson AJ, Govindan B (2015). Secondary metabolite from *Ganoderma*. Phytochemistry.

[3] Shiao MS (2003). Natural products of the medicinal fungus *Ganoderma lucidum*: occurrence, biological activities, and pharmacological functions. Chem. Rec..

[4] Chen S (2012). Genome sequence of the model medicinal mushroom *Ganoderma lucidum*. Nat. Commun..

[5] Mu D (2012). The development and application of a multiple gene co-silencing system using endogenous URA3 as a reporter gene in *Ganoderma lucidum*. PLoS One.

[6] Hirotani M, Asaka I, Furuya T (1990). Investigation of the biosynthesis of 3α-hydroxy triterpenoids, ganoderic acids T and S, by application of a feeding experiment using [1,2-^13^C_2_] acetate. J. Chem. Soc. Perkin. Trans..

[7] Shiao MS (1992). Triterpenoid natural products in the fungus *Ganoderma lucidum*. J. Chin. Chem. Soc..

[8] Shang CH, Shi L, Ren A, Qin L, Zhao MW (2010). Molecular cloning, characterization, and differential expression of a lanosterol synthase gene from *Ganoderma lucidum*. Biosci. Biotechnol. Biochem..

[9] Zhao MW (2007). Cloning and characterization of squalene synthase (SQS) gene from *Ganoderma lucidum*. J. Microbiol. Biotechnol..

[10] Xu JW, Zhao W, Zhong JJ (2010). Biotechnological production and application of ganoderic acids. Appl. Microbiol. Biotechnol..

[11] Yuan YJ, Li C, Hu ZD, Wu JC, Zeng AP (2002). Fungal elicitor-induced cell apoptosis in suspension cultures of *Taxus chinensis* var. *mairei* for taxol production. Process. Biochem..

[12] Wang JW, Xia ZH, Tan RX (2002). Elicitation on artemisinin biosynthesis in *Artemisia annua* hairy roots by the oligosaccharide extract from the endophytic *Colletotrichum* sp. B501. Acta. Bot. Sinica.

[13] Zhao J, Fujita K, Sakai K (2007). Reactive oxygen species, nitric oxide, and their interactions play different roles in *Cupressus lusitanica* cell death and phytoalexin biosynthesis. New. Phytol..

[14] You BJ (2013). A novel approach to enhancing ganoderic acid production by *Ganoderma lucidum* using apoptosis induction. PLoS One.

[15] Rollins JA, Dickman MB (1998). Increase in endogenous and exogenous cyclic AMP levels inhibits sclerotial development in *Sclerotinia sclerotiorum*. Appl. Environ. Microbiol..

[16] Phillips AJ, Crowe JD, Ramsdale M (2006). Ras pathway signaling accelerates programmed cell death in the pathogenic fungus *Candida albicans*. Proc. Natl. Acad Sci. USA.

[17] Lee YH, Dean RA (1993). cAMP regulates infection structure formation in the plant pathogenic fungus *Magnaporthe grisea*. Plant Cell.

[18] You BJ (2012). Effect of solid-medium coupled with reactive oxygen species on ganoderic acid biosynthesis and MAP kinase phosphorylation in *Ganoderma lucidum*. Food. Res. Int..

[19] Elmore S (2007). Apoptosis: a review of programmed cell death. Toxicol. Pathol..

[20] Meier P, Finch A, Evan G (2000). Apoptosis in development. Nature.

[21] Reape TJ, McCabe PF (2008). Apoptotic-like programmed cell death in plants. New Phytol..

[22] Fröhlich KU, Fussi H, Ruckenstuhl C (2007). Yeast apoptosis - from genes to pathways. Semin. Cancer Biol..

[23] Madeo F (2004). Apoptosis in yeast. Curr. Opin. Microbiol..

[24] Xu YN, Zhong JJ (2012). Impacts of calcium signal transduction on the fermentation production of antitumor ganoderic acids by medicinal mushroom *Ganoderma lucidum*. Biotechnol. Adv..

[25] Zhang WX, Zhong JJ (2010). Effect of oxygen concentration in gas phase on sporulation and individual ganoderic acids accumulation in liquid static culture of *Ganoderma lucidum*. J. Biosci. Bioeng..

[26] Xu YN, Xia XX, Zhong JJ (2013). Induced effect of Na^+^ on ganoderic acid biosynthesis in static liquid culture of *Ganoderma lucidum* via calcineurin signal transduction. Biotechnol. Bioeng..

[27] Xu YN, Xia XX, Zhong JJ (2014). Induction of ganoderic acid biosynthesis by Mn^2+^ in static liquid cultivation of *Ganoderma lucidum*. Biotechnol. Bioeng..

[28] Mu D (2014). Functions of the nicotinamide adenine dinucleotide phosphate oxidase family in *Ganoderma lucidum*: an essential role in ganoderic acid biosynthesis regulation, hyphal branching, fruiting body development, and oxidative-stress resistance. Environ. Microbiol..

[29] Studt L, Humpf HU, Tudzynski B (2013). Signaling governed by G proteins and cAMP is crucial for growth, secondary metabolism and sexual development in *Fusarium fujikuroi*. PLoS One.

[30] Choi YE, Xu JR (2010). The cAMP signaling pathway in *Fusarium verticillioides* is important for conidiation, plant infection, and stress responses but not fumonisin production. Mol. Plant. Microbe Interact..

[31] Shimizu K, Keller NP (2001). Genetic involvement of a cAMP-dependent protein kinase in a G protein signaling pathway regulating morphological and chemical transitions in *Aspergillus nidulans*. Genetics.

[32] Miyake T, Zhang MY, Kono I, Nozaki N, Sammoto H (2006). Repression of secondary metabolite production by exogenous cAMP in *Monascus*. Biosci. Biotechnol. Biochem..

[33] García-Martínez J, Adám AL, Avalos J (2012). Adenylyl cyclase plays a regulatory role in development, stress resistance and secondary metabolism in *Fusarium fujikuroi*. PLoS One.

[34] McConkey DJ, Orrenius S, Jondal M (1990). Agents that elevate cAMP stimulate DNA fragmentation in thymocytes. J. Immunol..

[35] Myklebust JH (1999). Activation of the cAMP signaling pathway increases apoptosis in human B-precursor cells and is associated with downregulation of Mcl-1 expression. J. Cell Physiol..

[36] Roze LV, Linz JE (1998). Lovastatin triggers an apoptosis-like cell death process in the fungus *Mucor racemosus*. Fungal Genet. Biol..

[37] Gourlay CW, Ayscough KR (2006). Actin-induced hyperactivation of the Ras signaling pathway leads to apoptosis in *Saccharomyces cerevisiae*. Mol. Cell Biol..

[38] Leadsham JE, Kotiadis VN, Tarrant DJ, Gourlay CW (2010). Apoptosis and the yeast actin cytoskeleton. Cell Death Differ..

[39] Ladilov Y, Appukuttan A (2014). Role of soluble adenylyl cyclase in cell death and growth. Biochim. Biophys. Acta..

[40] Valsecchi F, Ramos-Espiritu LS, Buck J, Levin LR, Manfredi G (2013). cAMP and mitochondria. Physiology (Bethesda).

[41] De Rasmo D, Signorile A, Papa F, Roca E, Papa S (2010). cAMP/Ca^2+^ response element-binding protein plays a central role in the biogenesis of respiratory chain proteins in mammalian cells. IUBMB Life.

[42] Leadsham JE, Gourlay CW (2010). cAMP/PKA signaling balances respiratory activity with mitochondria dependent apoptosis via transcriptional regulation. BMC Cell Biol..

[43] Giussani P, Tringali C, Riboni L, Viani P, Venerando B (2014). Sphingolipids: key regulators of apoptosis and pivotal players in cancer drug resistance. Int. J. Mol. Sci..

[44] Rego A (2014). The yeast model system as a tool towards the understanding of apoptosis regulation by sphingolipids. FEMS Yeast Res..

[45] Wada T, Penninger JM (2004). Mitogen-activated protein kinases in apoptosis regulation. Oncogene.

[46] Cuadrado A, Nebreda AR (2010). Mechanisms and functions of p38 MAPK signalling. Biochem. J..

[47] Duronio V (2008). The life of a cell: apoptosis regulation by the PI3K/PKB pathway. Biochem. J..

[48] Xu JW, Xu YN, Zhong JJ (2010). Production of individual ganoderic acids and expression of biosynthetic genes in liquid static and shaking cultures of *Ganoderma lucidum*. Appl. Microbiol. Biotechnol..

[49] Ren A (2010). Methyl jasmonate induces ganoderic acid biosynthesis in the basidiomycetous fungus *Ganoderma lucidum*. Bioresour. Technol..

[50] Liang CX (2010). Enhanced biosynthetic gene expressions and production of ganoderic acids in static liquid culture of *Ganoderma lucidum* under phenobarbital induction. Appl. Microbiol. Biotechnol..

[51] Ren A (2014). Transcript and metabolite alterations increase ganoderic acid content in *Ganoderma lucidum* using acetic acid as an inducer. Biotechnol. Lett..

[52] Zhao W, Xu JW, Zhong JJ (2011). Enhanced production of ganoderic acids in static liquid culture of *Ganoderma lucidum* under nitrogen-limiting conditions. Bioresour. Technol..

[53] Ren A (2013). Profiling and quantifying differential gene transcription provide insights into ganoderic acid biosynthesis in *Ganoderma lucidum* in response to methyl jasmonate. PLoS One.

[54] You BJ (2012). Enhanced production of ganoderic acids and cytotoxicity of *Ganoderma lucidum* using solid-medium culture. Biosci. Biotechnol. Biochem..

[55] Grabherr MG (2011). Full-length transcriptome assembly from RNA-Seq data without a reference genome. Nat. Biotechnol..

[56] Robinson MD, McCarthy DJ, Smyth GK (2010). edgeR: a Bioconductor package for differential expression analysis of digital gene expression data. Bioinformatics.

[57] Kanehisa M, Sato Y, Kawashima M, Furumichi M, Tanabe M (2016). KEGG as a reference resource for gene and protein annotation. Nucleic Acids Res..

[58] Kanehisa M, Goto S (2000). KEGG: Kyoto Encyclopedia of Genes and Genomes. Nucleic Acids Res..

