# Supplementary material for: Induction of apoptosis and ganoderic acid biosynthesis by cAMP signaling in Ganoderma lucidum
Source: Sci Rep. 2017 Mar 23;7:318. doi: 10.1038/s41598-017-00281-x (PMC5428012; doi:10.1038/s41598-017-00281-x)
Supplement: Supplementary file 1 — Supplementary information [file 41598_2017_281_MOESM1_ESM.pdf]

## Supplementary information -SREP-15-33255C

### Induction of apoptosis and ganoderic acid biosynthesis by cAMP signaling in *Ganoderma lucidum*

Bang-Jau You[1,†,*], Ni Tien[2,†], Miin-Huey Lee[3], Bo-Ying Bao[4], Yih-Shyuan Wu[1], Tsung-Chi Hu[1], and Hong-Zin Lee[4,*]

[1]Department of Chinese Pharmaceutical Sciences and Chinese Medicine Resources, China Medical University, Taichung, Taiwan

[2] Department of Laboratory Medicine, China Medical University Hospital, Taichung, Taiwan

[3] Department of Plant Pathology, National Chung-Hsing University, Taichung, Taiwan

[4]Department of Pharmacy, China Medical University, Taichung, Taiwan

[†]These authors contributed equally to this work.

[*]Correspondence: bangjau@mail.cmu.edu.tw (B.J. You), hong@mail.cmu.edu.tw (H.Z. Lee)

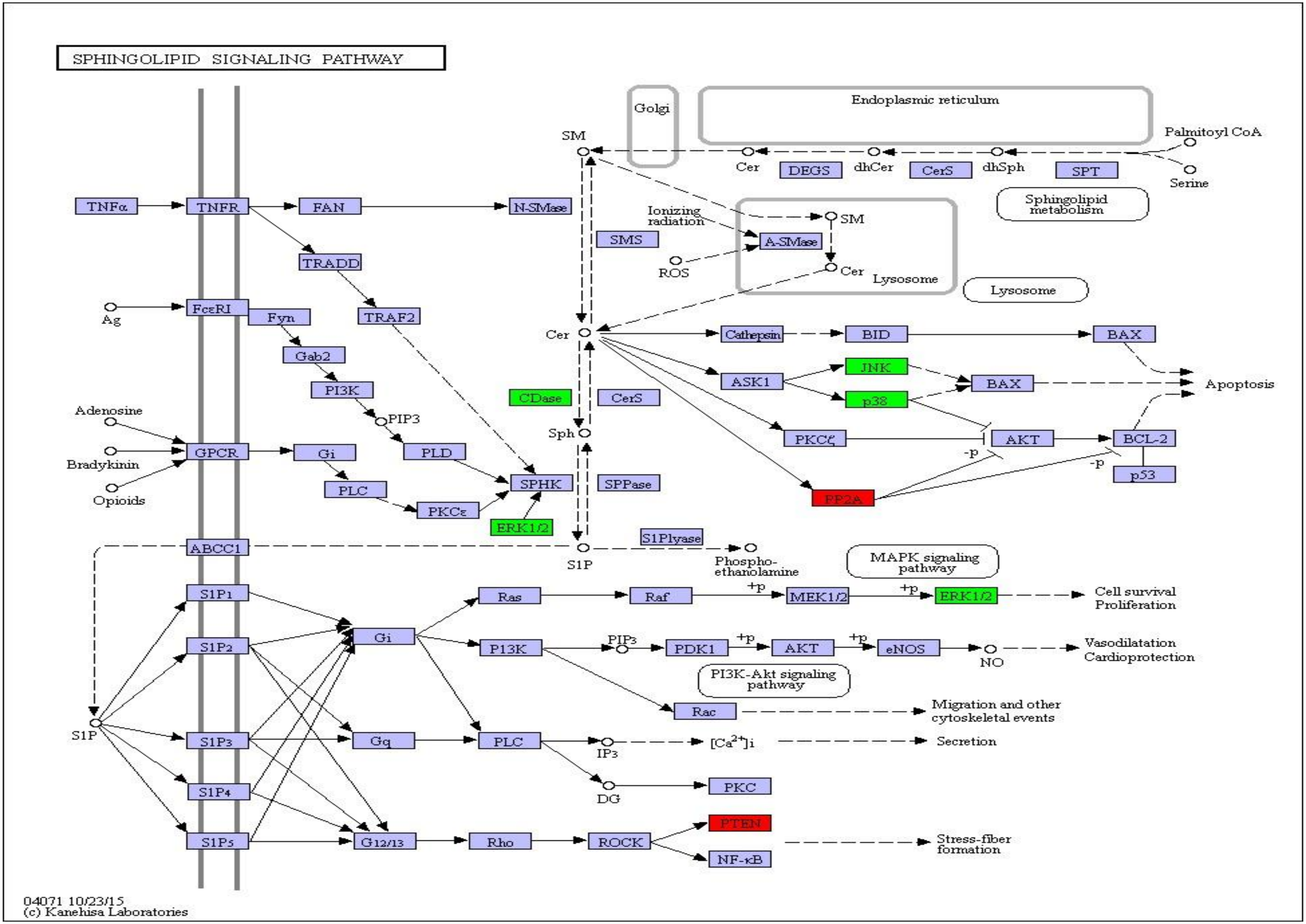


**Supplementary Figure 1**

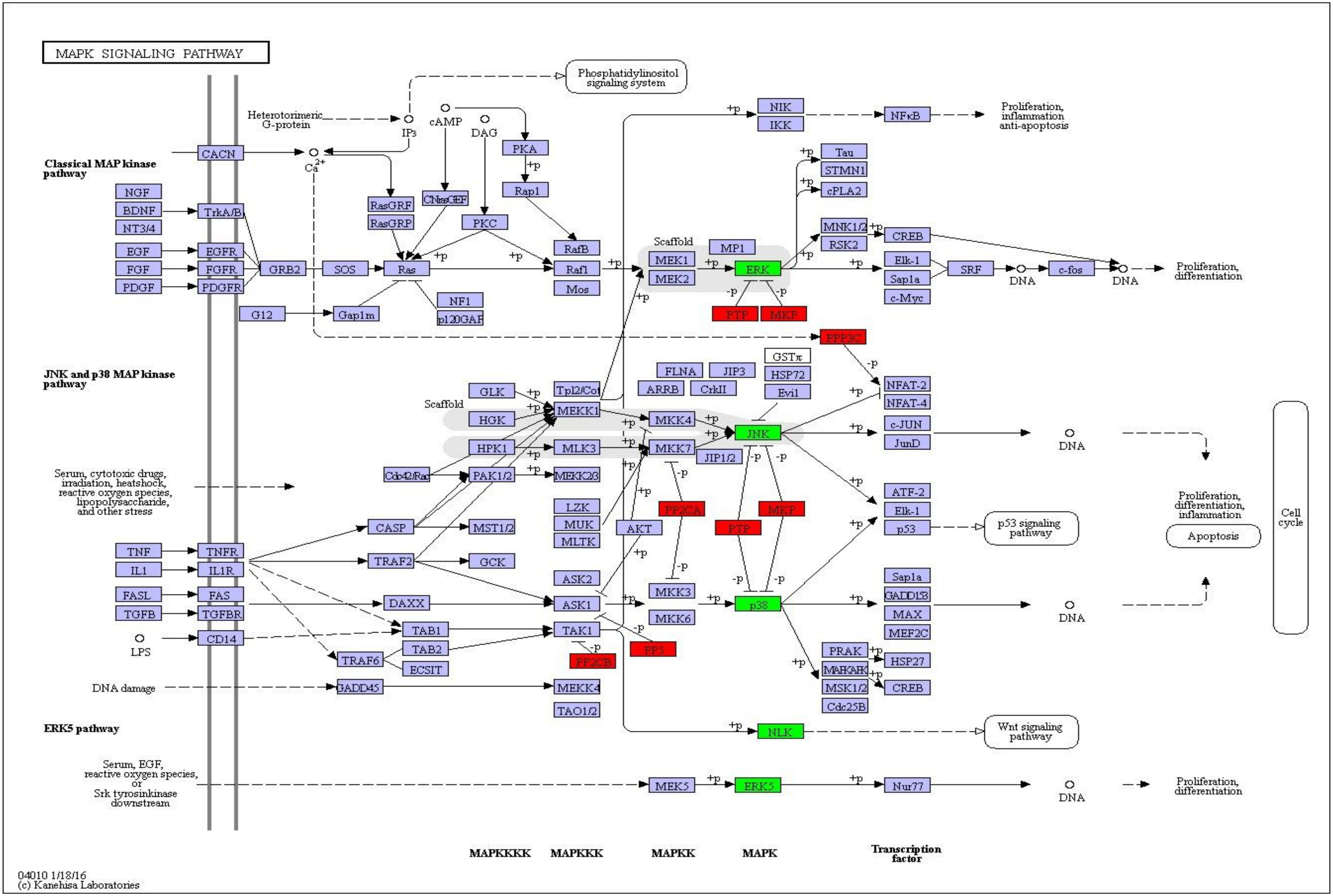


**Supplementary Figure 2**

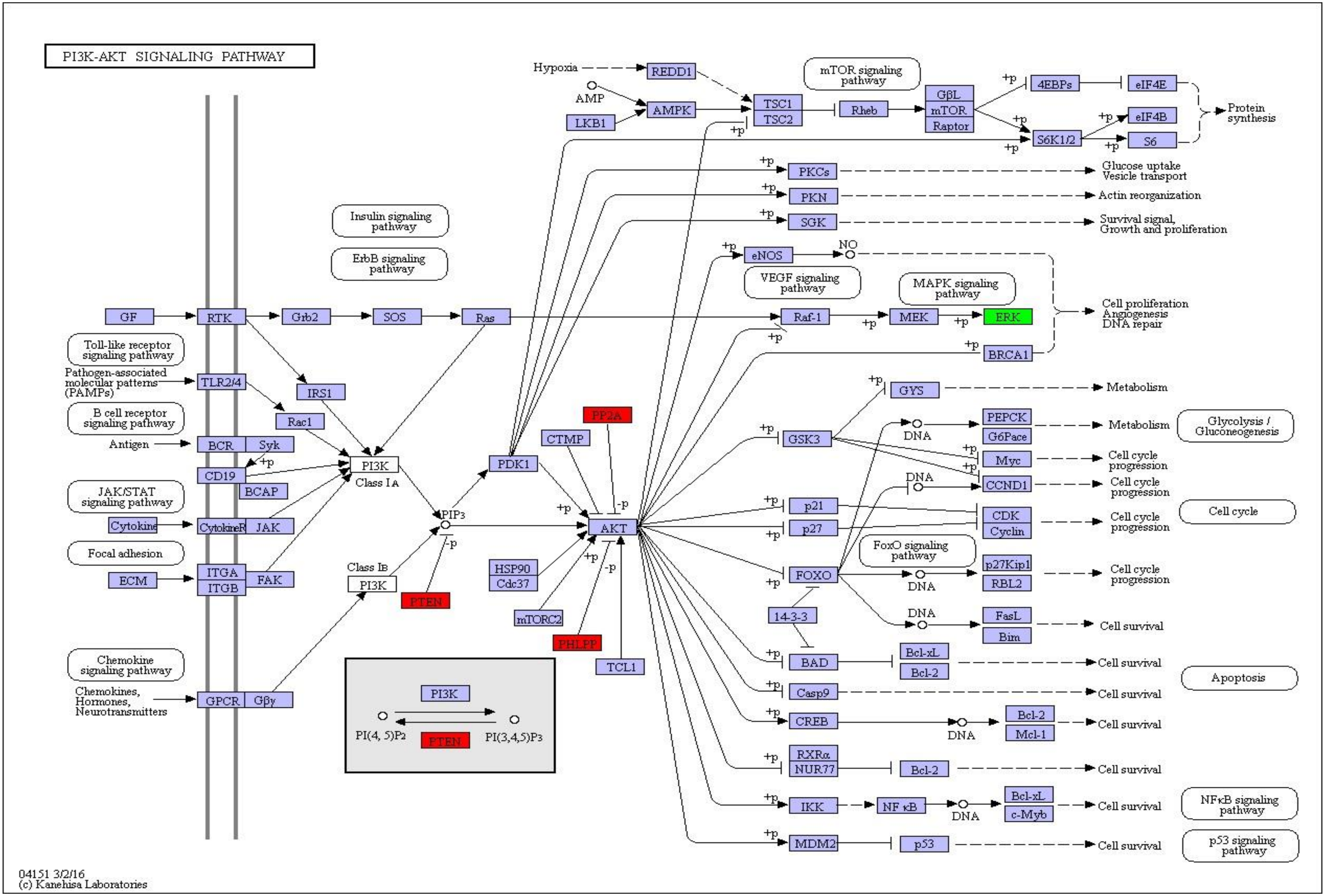


**Supplementary Figure 3**

## Supplementary Table legends

**Supplementary Table 1. Sequence annotation and gene expression in the control and cAMP-treated *Ganoderma lucidum* mycelium.** LG-Ctrl_count: reads count of control mycelium; LG-IBMX-CAM_count: reads count of mycelium incubated with cAMP; LG-Ctrl_fpkm: normalized FPKM value of control mycelium; LG-IBMX-CAM_fpkm: normalized FPKM value of mycelium incubated with cAMP; logFC: log2 fold change; logCPM: log2 total reads per million; FDR: False Discovery Rate.

**Supplementary Table 2. Transcripts of oxidative phosphorylation complexes showing enhanced expression in *Ganoderma lucidum* after treatment with cAMP.**

## Supplementary Figure legends

**Supplementary Figure 1. Heat map of differentially expressed genes in the sphingolipid signaling pathway.** The red and green boxes represent the genes that were upregulated and downregulated in response to cAMP, respectively.

**Supplementary Figure 2. Heat map of differentially expressed genes in the MAPK signaling pathway.** The red and green boxes represent the genes that were upregulated and downregulated in response to cAMP, respectively.

**Supplementary Figure 3. Heat map of differentially expressed genes in the PI3K-AKT signaling pathway.** The red and green boxes represent the genes that were upregulated and downregulated in response to cAMP, respectively.
